# An economic evaluation of the effectiveness of telemedicine in hematooncology

**DOI:** 10.1371/journal.pone.0291143

**Published:** 2023-11-14

**Authors:** Marian Lebiedzik, Kamila Turečkova, Ingrid Majerova, Jan Nevima, Katarína Hradská, Tereza Popková, Michaela Skořupová, Roman Hájek

**Affiliations:** 1 Department of Economics and Public Administration, Silesian University in Opava, School of Business Administration in Karvina, Karvina, Czech Republic; 2 Department of Haematooncology, University Hospital Ostrava and Faculty of Science, University of Ostrava, Ostrava, Czech Republic; 3 Department of Haematooncology, University Hospital Ostrava and Faculty of Medicine, University of Ostrava, Ostrava, Czech Republic; The University of Texas MD Anderson Cancer Center, UNITED STATES

## Abstract

The article examines the effectiveness of remote monitoring and the evaluation of facts about patients with hematologic malignancies using telemedicine based on SMART technologies. The project was carried out in the Department of Haematooncology of the University Hospital Ostrava. Its objective was to test the efficacy of telemedicine in the treatment of patients with blood cancer. The cost-benefit analysis method was used to evaluate effectiveness, which also confirmed the feasibility of using this method to evaluate the costs and benefits of implementing specific medical projects. The conducted analysis demonstrated the effectiveness of using telemedicine procedures in the treatment of these patients, both in terms of quantifiable and non-quantifiable impacts on the Czech Republic’s health system. This was mainly due to a large shortening of the length of the hospitalisation period for patients with problems whose deterioration was discovered by remote monitoring and their treatment could begin promptly. The shortening of the hospitalization period was achieved by around 40%. As a result, the complexity of treatment has been greatly reduced, benefiting both the hospital and, most importantly, the patient. With this prevention, the patient’s chances of dying are reduced, as he or she is less likely to develop severe septic diseases. The total average financial savings of the Czech Republic’s entire health care system for a patient who does not become septic due to a delayed response to deteriorating health only in hospitalisation, treatment, and medications is approximately USD 2,800.

## Introduction

In the Czech Republic, the number of patients with hematologic cancer is steadily increasing. According to data from the Czech Institute of Health Information and Statistics, there were 20,600 patients with hematologic tumours in our country in 2005, 27,100 in 2014, 32,300 in 2016, and 34,600 in 2018 representing a 57% increase in ten years. These patients are vulnerable to a variety of complications related to the disease or subsequent treatment. The annual increase in the incidence of hematooncological diseases in the general population, combined with an increase in the proportion of older age groups, is putting pressure on the availability of health services, with an increase in the number of outpatient visits and acute hospitalisations expected [1].

Hematooncology patients are subjected to arduous oncological treatment, which always results in a decrease in the number of individual blood cells in the blood count. These patients are vulnerable to a variety of complications related to the disease or subsequent treatment. As patients have weak immunity, the course of the disease and treatment are frequently complicated by various infections. These are frequent infections that do not occur in healthy people, or they are common diseases that are difficult to treat in these patients. Although chemotherapy-treated hematooncological patients move to outpatient care, the speed with which the patient enters the medical facility is a critical factor for success.

Infections, particularly febrile neutropenia, are one of the most serious complications of anticancer treatment, with significant morbidity, mortality, and, not least, significant financial costs of care. Febrile neutropenia is defined as a body temperature value of 38.0°C or higher that lasts more than an hour in patients with an absolute neutrophil count of less than 0.5 x 109 / l or an expected decrease of less than 0.5 x 109 / l. [2, 3]. Patients with blood tumours are generally considered to be more at risk than patients with solid tumours due to the longer duration of neutropenia = reduced immune system [3]. Morbidity and mortality rates reach 20–30% and 10%, respectively [2, 4]. Time, specifically the time to administer broad-spectrum antibiotics, is the most important factor in the development of an infection that affects more than 80% of patients during treatment [2, 3, 5]. Septic shock, multiorgan failure, and premature death can be avoided if this potentially fatal condition is detected early, and anti-infective treatment is administered promptly.

At the same time, modern and effective treatment is prohibitively expensive, particularly when complications arise. In these cases, it is critical to detect any deterioration in the patient’s health and to initiate effective treatment as soon as possible. Therefore, this approach often does not require hospitalisation of the patient, which is directly related to reducing the financial costs of its implementation [6].

In this context, there is the option of using telemedicine and cutting-edge technologies to provide health care in the field of hematooncology. It must be said that the use of telemedicine in the field of hemato-oncology is currently not as widespread as it is in other medical fields, for example, cardiology. Therefore, experience with the use of telemedicine tools in hematology. A certain positive impulse in this direction was the epidemic of COVID-19, when there is a sharp increase in telemedicine in this field as well [7]. Telemedicine, like digitisation and telemetry, is a response of healthcare to technological development and innovation, with implications for the treatment system that aims to connect patients and physicians remotely [8]. Connecting these three modern modes of "communication" is highly effective at all levels and generates significant benefits for all stakeholders in this system [9]. The Department of Heamatooncology, University Hospital Ostrava, is one of the workplaces that uses telemedicine to care for their patients. This Clinic has recently implemented the project "Telemedicine in hematooncology—progress in remotely monitoring patients and increasing the safety of treatment in patients with blood tumours [10].

The purpose of this paper is to assess the efficacy of telemedicine in the treatment of blood cancer. To achieve the set goal, the cost-benefit analysis (CBA) method appears to be appropriate, as it is distinguished by its uniform application across scientific disciplines [9] and can be considered the most appropriate methodological ratio approach tool [11] in decision-making processes [12].

CBA is an analytical tool and comparative approach [13] used to evaluate, in particular, activities and operations of the public sector. The CBA is based on an analysis of all costs and benefits, both implicit and explicit, that quantifies the impact of investments on the company. The net socioeconomic surplus (benefit) of the company or a part of it serves as the evaluation criterion. On the one hand, we have all of the economic and social costs, expressed numerically or financially, and on the other hand, we have all of the benefits that increase the company’s welfare. We choose one that maximises the difference between the costs and benefits of the analysed activity by aggregating both types of impact, negative and positive, or by comparing individual variants with each other, or the activity should be undertaken if the expected benefits exceed the expected costs. The purpose of the CBA is to promote a more efficient allocation of resources and to demonstrate its value to the company [12, 14–17]. The emergence of the CBA coincides with the emergence of the valuation of public activities and projects [12, 14–17], which distinguishes it from other analyses that emerged in the second half of the twentieth century [18].

The CBA is based on the premise that the value of all negative and positive impacts is measurable, both directly and indirectly, in monetary units. Meeting this assumption and expressing all the benefits and negative impacts in cash flows is advantageous and allows the CBA to be applied to almost any activity. Concurrently, it is a problematic point in the analysis as many input and output effects are intangible or non-financial in nature, and determining individual effects at all is difficult. Together with the description and monetary expression of the effects, the definition of entities that will be affected by the anticipated implemented activity (solution) and in what manner [9] is closely related.

Financial and economic analysis are critical components of the CBA analysis. The goal of financial analysis is to determine the project’s financial efficiency. Financial analysis consists primarily of the quantification of investments, the calculation of project costs and revenues, the planning of the project cash flows, and the quantification of financial efficiency indicators [14].

Only explicit financial costs and revenues are considered decisive in this analysis. The most critical factor is efficiency. Economic analysis examines activities from a social perspective and evaluates them using indicators similar to those used in financial analysis. In addition, the basic criterion for assessing the suitability of activity is fairness and, in particular, the impact on the overall benefit of the company. The assessed activity should primarily contribute to the growth of social welfare. The main financial indicators or economic analysis include (1) net present value, or economic net present value (NPV or ENPV); (2) internal rate of return, or economic internal rate of return (IRR or ERR) and (3) benefit-cost ratio (BCR or B / C), or cost-benefit ratio (CBR or C / B) [9].

The content of the presented paper overlaps the medical professions of hematooncology and social sciences, particularly economics (economic analysis), and determines whether the use of telemedicine is more effective in comparison to current medical practise through the CBA. The European Union has also declared the suitability of using the CBA for project effectiveness decisions, describing it as an analytical tool to evaluate investment decisions [14]. At the same time, the feasibility of using the CBA to assess the introduction of new medical procedures will be investigated.

## Materials and methods

### Procedure

Actively being treated patients with blood tumours are included in the project with longer expected neutropenia and therefore a high risk of infection. Target diagnoses include a) acute leukaemias (myeloid, promyelocytic, lymphoblastic)—during active treatment, including the post-transplant period; b) malignant lymphomas (Hodgkin’s and non-Hodgkin’s)—intensive regimens in primary therapy, salvage regimens in relapse, the period after autologous hematopoietic transplantation and after CAR (chimeric antigen receptor) -T cell treatment; c) multiple myeloma—intensive regimens, regimens with anti-CD38 antibody, the period after autologous and allogeneic hematopoietic transplantation; d) chronic lymphocytic leukaemia—regimens containing fludarabine or obinutuzumab in first line therapy; e) myelodysplastic syndrome—with concomitant neutropenia; f) other—for example very severe aplastic anaemia after application of anti-thymocyte globulin or hairy cell leukaemia after treatment with cladribine.

A doctor involved in the project contacts each potentially suitable candidate, usually during hospitalisation at the beginning of a specific line of treatment or as part of an outpatient check-up during treatment. If the patient expresses an interest, informed consent is signed by him/her, and the initial education of the parties to the measurement regime and the use of measuring instruments is performed.

Each participant is equipped with a digital pressure gauge (MD2020, MD4781, Beurer BM 85) and a non-contact infrared thermometer (Rycom JXB-182), both of which are Bluetooth-enabled and connected to a mobile hub (mobile phone). In the privacy of their own homes, patients check their blood pressure/ pulse and body temperature once a day (between 6:00 and 10:00 am), or twice a day (between 6:00 and 10:00 am, 5:00 and 9:00 pm?), and at any time if there is subjective deterioration. Measured values are sent to a secure server, which authorised medical personnel can access from any device with an Internet connection. Values that are outside the specified range are immediately notified by SMS, allowing the doctor to quickly assess the situation, contact the patient (via SMS directly via the portal or by phone), and recommend further action.

Two types of alarms can be set: warning and critical. A warning alarm is triggered when body temperature exceeds 37.0°C, and a critical alarm is triggered when the body temperature exceeds 37.9°C. The critical values of pressure and pulse are as follows: systolic blood pressure (STK) <90mmHg, diastolic blood pressure (DTK) <50mmHg or> 90mmHg, and pulse <40 / min or> 130 / min. We primarily monitor temperature increases, but the pressure and pulse values are considered complementary in the decision-making process, as hypotension with tachycardia could indicate serious infection.

Telemedicine and telemonitoring allow physicians to monitor body temperature and blood pressure in home care. The patient who undergoes outpatient treatment has a lower risk of infection with multiresistant nosocomial strains, has a higher quality of life, and, finally, pays a lower treatment cost. Telemonitoring allows for immediate consultation of the patient’s current state of health with the doctor, allowing adequate assistance to the patient in detecting fever and changes in blood pressure. This measurement has the potential to prevent the development of sepsis, which can be fatal to the patient.

### Data collection

The implementation of the project is associated with considerable costs for the acquisition of the necessary equipment and personnel. Table 1 summarises the total costs associated with the implementation of the project. According to the table above, the monthly cost of monitoring one patient is USD 105 or USD 4 per day.

**Table 1:** Telemedicine project implementation costs.

Item	Price (in USD)	Quantity	Period (M, D)	Price (in USD)
Pressure gauge	0,68	30	1170 days	23 696
Non-contact thermometer	0,40	30	1170 days	13 939
Data transmission device (mobile HUB)	0,52	30	1170 days	18 121
Data transmission 3 GB incl. servers	2,58	30	39 months	3 020
Price of a telemed. service partner	0,52	30	1170 days	18 121
Labour costs (2 doctors)	595,69/doctor	2	39 months	46 464
Total costs for 39 months of monitoring		30		12 3361
Costs for 1 month of monitoring		30		3 163
Costs per patient for 1 month of monitoring		1		105
Costs per patient for 1 day of monitoring		1		4

Source: UHO, our own calculation

The analysis includes two groups of patients that were monitored and evaluated during 2019 and 2020.

The first group consists of patients who participated in the telemedicine project and whose health status was monitored and consulted remotely during the project’s implementation. This was relatively successful in preventing a situation where they arrived late at the hospital and became septic as their current state of health deteriorated. This group included seven patients. Table 2 summarises the hospital’s payments to insurance companies and the costs of their treatment.

**Table 2:** Basic parameters of treatment of project patients (1^st^ group of patients) who did not develop sepsis due to telemedicine.

Patient no.	Number of hospitalization days	Costs of hospitalization and treatment in USD	Costs of drugs in USD	Costs of medical procedures in USD	DRG (payments in USD)
1	8	4882	1764	3118	3314
2	6	2908	494	2414	2535
3	6	2909	513	2396	2535
4	10	6892	1965	4926	2475
5	4	1655	124	1531	3879
6	8	4993	1264	3728	3632
7	5	5993	2541	3452	3187
Total	47	30232	8666	21565	21557
Average per patient	6,7	4319	1238	3081	3080
Average cost and DRG per patient and per day of treatment		645	185	460	460

Source: UHO, our own calculation

According to Table 2, the average length of hospital treatment for these patients is 6.7 days. The average cost of treating one patient is USD 4,319, while the costs of each patient vary greatly depending on their health condition. On the contrary, the differences in insurance companies’ payments to the hospital for individual patients’ treatment are significantly lower, amounting to an average of USD 3,080 per patient. As a result, it is clear that the cost of treating patients exceeds the reimbursement of insurance companies, and it is in the hospital’s best interest to reduce treatment costs, provided that the quality of care provided does not suffer. It is worth noting that the hospital paid an average of USD 185 per patient for one day of his/her hospital treatment. Although remote monitoring patients are less likely to develop a serious medical condition leading to death than other patients with the same diagnosis, we do report one patient who died in this group, not due to infectious complications but due to the progression of hematologic malignancy.

The second group consists of patients who did not participate in the telemedicine project and thus frequently arrived at the hospital in a more serious condition, reaching the stage of sepsis. The control group included 30 patients, two of whom died.

Treatment in this group lasted an average of 11.2 days (see Table 3). The average cost of hospitalisation for a patient was USD 6,749, with insurance companies paying an average of USD 4,361. Even in this case, the hospital is compelled to pay a surcharge for patient care. The average supplementary charge for one-day treatment and hospitalisation of the patient is then USD 214, which is higher than in the first group of patients. This comparison demonstrates that it is in the best interests of the medical facility if treated patients do not progress to the stage of sepsis. As stated previously, remote patient monitoring should help with this.

**Table 3:** Basic parameters of treatment of non-project patients (2^nd^ group of patients) who developed sepsis.

Patient no.	Number of hospitalization days	Costs of hospitalization and treatment in USD	Costs of drugs in USD	Costs of medical procedures in USD	DRG (payments in USD)
1	8	3068	204	2865	3347
2	10	7858	848	7010	3244
3	9	5437	1605	3832	4411
4	11	7236	1384	5852	3743
5	7	2181	526	1655	3347
6	14	3496	893	2603	2355
7	7	2436	532	1904	3186
8	6	4034	1420	2613	3479
9	23	4597	313	4284	1993
10	8	4232	956	3276	3400
11	19	14451	6220	8232	8388
12	6	3641	1034	2606	3474
13	8	5849	2402	3448	3436
14	12	3271	562	2709	3501
15	7	5234	1008	4226	3623
16	8	5841	1463	4378	4263
17	11	9820	3571	6249	6458
18	8	3876	491	3385	1860
19	10	5031	546	4485	3347
20	7	2728	494	2234	3501
21	6	2539	569	1971	8499
22	9	5176	1530	3645	3944
23	6	5897	1974	3923	4584
24	11	7375	2432	4943	4720
25	6	3408	967	2441	3411
26	42	34026	16363	17663	14639
27	10	8286	2682	5605	4006
28	17	10961	3940	7022	4954
29	14	9397	2845	6552	4129
30	15	11097	2123	8974	3585
Total	335	202478	61895	140583	130828
Average per patient	11,2	6749	2063	4686	4361
Average cost and DRG per patient and per day of treatment		604	185	420	391

Source: UHO, our own calculation

### Methods used

The analysis of the experimental verification of the application of telemedicine in hematooncology, the main purpose of which is remote patient monitoring, is based directly on primary data from the Clinic of Hematooncology of the University Hospital Ostrava. Patient data was collected in 2019 and 2020 and reflect the basic treatment parameters of two groups of sick people that were compared. The first group of patients consists of those who participated in the telemedicine project (there were seven of them), while the second group consists of patients who were treated using the standard approach (a total of 30 people). According to the CBA, the telemedicine application can be described as (1) an evaluated project in relation to the second option, and (2) a zero variant. Our CBA is limited by the evaluation of indirect effects: a thorough financial analysis was performed, which was supplemented by a list of explicit effects of cost and benefit nature. If these effects could be quantified in monetary units, our research could be supplemented by the CBA’s second component, economic analysis. The patient’s input data included information on the number of hospitalisation days, the hospitalisation cost, the DGR revenues (payments from the public health system), and the costs of implementing a telemedicine project.

The efficacy of both options, the evaluated telemedicine project, and the null variant was determined by a simple difference in the relevant values of the indicators in both studied groups of patients, as well as an evaluation of total treatment costs in relation to benefits through a C / B coefficient that measures the sum of total costs to the sum of total benefits. In our case, we abstract from both inflation and the discount rate, as well as the time horizon, the project’s lifespan. For the C / B coefficient (ratio), it is true that the lower its value, the more efficient (suitable) the investment project from a financial analysis standpoint. Although positive values of the C / B indicator represent the project’s financial disadvantage, the absence of social costs and benefits, and vice versa, must be reflected because monetary costs exceed revenues. In the following two chapters, all input information, calculations, analytical procedures, and conclusions of the evaluation performed of the experimental verification of the application of telemedicine in relation to the current state are presented.

## Results

As part of this experimental verification, analysis and evaluation of the benefits and costs of the telemedicine project were performed from the point of view of all stakeholders, including the health system (including the state or health insurance companies), hospitals as care providers and project implementers, and, finally, a patient (and his close relatives) as a client of the healthcare system and the hospital. According to the cost-benefit analysis method, the "costs" group included the wages costs of the project members involved in the development of the navigation system, the purchase of smart equipment, and also maintenance for the life of the system. The "benefits" group included time savings for all hospital staff who participated in the first step of the project (the respondents to the questionnaire survey).

The analysis is based on a comparison of the benefits and costs of the so-called investment and zero variants. The situation in which the hospital does not implement (invest) in the aforementioned telemedicine project is considered a zero variant. In this case, it will be based on the costs and revenues values associated with the hospitalisation and treatment of the second group of patients mentioned above. In the case of the investment variant, the hospital provides continuous monitoring and counselling to the first group of patients through telemedicine. The investment covers both the purchase of telemedicine equipment and the wage costs associated with the medical provision of this service.

First, the benefits of telemedicine are considered in terms of the operation of the healthcare system. As the project is funded by the hospital, the costs imposed by the investment variant on the state, i.e., the health insurance company, are zero. In contrast, due to the significantly shorter hospitalisation period of patients in the first group compared to patients in the second group, telemedicine generates total savings of USD 2,430 per hospitalised patient who does not reach the stage of sepsis (the difference is 4.5 days). This sum is sufficient to cover 23 months of patient monitoring.

The data in Table 4 show that when the total average cost of a patient’s hospitalisation and treatment of a patient is recalculated per day of this patient’s hospital stay, both the costs and insurance company payments that the hospital receives are higher in the first group of non-aseptic patients. However, this fact does not change the previously stated higher financial demands of treating septic patients, which are due to the significantly longer duration of treatment in the medical facility.

**Table 4:** Evaluation of total treatment costs of the first group of monitored patients (investment variant) and the second group of unmonitored patients (zero variant) from the point of view of the health care system.

	1^st^ group of patients	2^nd^ group of patients	Difference between the 1^st^ and 2^nd^ group of patients
The average number of days of hospitalisation	6,7	11,2	4,5
Average cost per hospitalisation day per patient in USD	645	604	-40
Average DRG per hospitalisation day per patient in USD	460	391	-69
Cost per patient per monitoring day in USD	4	0	-4
Average total hospitalisation savings per average patient who does not reach the stage of sepsis in USD			2 430
Number of months of monitoring covered by hospitalisation savings			23

Source: UHO, our own calculation

As a result of the implementation of telemedicine, payments for services related to the longer stay of patients in the hospital, and thus the complexity of care for these patients, are reduced. In some cases, this can reduce the overall length of treatment and the cost of paying for health benefits. Monitoring operations reflect an increase in the efficiency of the healthcare system and the quality of healthcare provided. It is probably reflected in the longer life expectancy and lower mortality of patients.

However, due to the different number of measurements within each patient group, this is difficult to demonstrate using the data available for each patient group.

Table 5 assesses the effects of patient monitoring on the hospital itself, which decided to implement the telemedicine project and is also responsible for the so-called investment costs. As stated above, the costs of acquiring and operating monitoring equipment, as well as the increased wage costs of physicians involved in monitoring implementation, total USD 105 per month.

**Table 5:** Evaluation of the total treatment costs of the first group of monitored patients (investment variant) and the second group of unmonitored patients (zero variant) from the hospital point of view.

	1^st^ group of patients	2^nd^ group of patients	Difference between the 1^st^ and 2^nd^ group of patients
The average number of days of hospitalisation	6,7	11,2	4,5
Average cost per hospitalisation day per patient in USD	645	605	-40
Average DRG per hospitalisation day per patient in USD	460	391	-69
Cost per patient per monitoring day in USD	4	0	-4
Average supplementary hospital charge for patient treatment (1 day of hospitalisation of 1 patient) in USD	185	214	29
Average total hospital savings for hospitalisation of one average patient who does not reach the stage of sepsis in USD	0	0	963
Number of months of monitoring covered by hospitalisation savings			9
C/B ratio	1,402	1,548	

Source: UHO, our own calculation

Even in hospitals, the reduction in hospitalisation period for patients participating in telemedicine is undeniably positive. The hospital can use the vacated beds, particularly those in intensive care, for other patients who require this care. After more than a year of dealing with the COVID-19 epidemic, it is clear that demand for these types of beds will be high for some time and will continue to be so even after the epidemic has subsided when post-Covid diagnosed patients or those whose care has been delayed due to coronavirus for a variety of reasons are most likely to use them. In most cases, the hospital’s operating costs will be reduced because of the shorter length of hospitalisation.

Even when simply comparing financial costs and DRG payments (revenues), the benefit of the telemedicine project is evident from the hospital’s perspective when the C / B ratio for one day of treatment of the monitored patient is lower than that of the unmonitored patient.

## Discussion

As stated above, the hospital is not fully reimbursed by the insurance company for the treatment of both groups of patients, and the hospital must cover a part of the actual hospitalisation and medication costs from its resources. If the hospital pays USD 214 for one patient with sepsis, the hospital treatment of the patient included in the monitoring project costs only USD 185. Although the difference may not appear to be significant at first glance, with a larger number of patients with this diagnosis, it may be a significant amount per year. After all, the average total hospital savings for hospitalisation of one average patient who does not reach the stage of sepsis is USD 963. This is the cost of running telemedicine monitoring for one patient for nine months.

In connection with the implementation of the telemedicine project, it is also important to note the non-financial benefits of improving the hospital’s image and positive public perception. The given project is appealing to the general public and the patients involved, and with adequate media coverage, the prestige of not only the Clinic for Hematooncology at UHO but also the hospital itself will undoubtedly rise.

The average annual hospital savings for the hospitalisation of a patient whose deterioration in health was detected in time thanks to monitoring and thus did not reach the stage of sepsis was stated to be USD 1,263. This is true if individuals in both groups are hospitalised once a year. However, the hospital is not the only one who saves money in these cases. Furthermore, the health insurance company saves the hospital USD 1,420 in its payments (see Table 6). The total average financial savings of the entire Czech Republic’s health care system for a patient who does not become septic due to a delayed response to deteriorating health only in hospitalisation, treatment, and medications is USD 2,443.

**Table 6:** Comparison of average annual costs and payments for the treatment of monitored and unmonitored patients.

	1^st^ group of patients	2^nd^ group of patients	Difference between the 1^st^ and 2^nd^ group of patients
The average number of days of hospitalisation	6,7	11,2	-
Average cost per hospitalization day per patient in USD	645	604	-40
Average DRG per day of hospitalisation for the patient in USD	460	391	-69
Cost per patient per monitoring day in USD	4	0	-4
Average annual total cost per year and patient in USD	4 322	6 765	2 443
Average annual total additional funding of the hospital for hospitalisation of 1 average patient who does not reach the stage of sepsis in USD	1 239	2 385	1 146
Costs per patient per year of monitoring in USD	1 265 (including wages) 789 (equipment only)		
Average total annual savings per hospitalisation of 1 average patient who does not reach the stage of sepsis in CZK		The healthcare system (insurance companies): 1 420	Hospitals: 1 146
The average return on investment		310 days 193 days (equipment only)	366 days 228 days (equipment only)

Source: UHO, our own calculation

Then it is possible to quantify the return on investment in the form of the acquisition of equipment required for the implementation of patient monitoring in the context of these figures. It is 288 days for a hospital, 193 days for health insurance companies, and only 105 days for the entire healthcare system (including hospitals).

If the costs include the annual wage costs of the doctors who perform the monitoring, the hospitals will recoup their investment within the first year of implementing the monitoring (return 366 days). After the first year, the hospital only pays the wage costs for the physicians, which is about 38% of the total average savings generated by not having to treat patients with sepsis. This savings is even more significant in the case of insurance companies, where the share of wage costs in savings is just 32%.

For illustration, the hospital monitors the health of 30 leukaemia patients over three years. Due to this monitoring, no patients will be hospitalised with sepsis throughout the period, but will be treated in a hospital once a year due to the deterioration of the health condition detected by telemedicine. In this case, the hospital that provides care saves USD 75,000 by funding monitoring through telemedicine. After accounting for the 135,000 saved by health insurance companies, the total financial social benefit for only thirty patients with the stated diagnosis over three years is approximately USD 210,000. Additional social benefits can be expected in the form of lower mortality and longer life expectancy for these patients, both of which are difficult to quantify and quantify.

Implementing the telemedicine project has immediate and significant consequences for patients, their families, and loved ones. In terms of financial quantification, costs or benefits are difficult to determine from their perspective. The cost to the patient or, in more complicated cases, to family members is the time and effort required to participate in the monitoring project. In particular, it is necessary to regularly monitor one’s health through temperature measurement, communication with the doctor, etc.

The obvious advantage of the patient in the early detection of deterioration of health and the need for hospitalisation is to shorten its time. As stated above, the average length of the hospitalisation period is reduced by 4.5 days. Telemonitoring also reduces the risk of death by preventing the patient from developing more serious medical conditions, particularly sepsis. In this context, the complexity of treatment and the subsequent duration of convalescence are reduced, as are the mental and physical strain associated with this treatment. Closely related to this is the shortening of the potential care time of family members associated with possible health complications for patients.

## Conclusion

The CBA confirmed that telemedicine and the procedures mentioned above are both effective in the treatment of patients with blood cancer. The UHO procedures implemented in this area have resulted in huge cost savings across the Czech Republic’s overall healthcare system, both on the part of public health insurance and the hospital itself. This was mainly due to a large shortening of the length of the hospitalisation period for patients with problems whose deterioration was discovered by remote monitoring and their treatment could begin promptly. The shortening of the hospitalisation period was achieved by around 40%. As a result, the complexity of treatment has been greatly reduced, benefiting both the hospital and, most importantly, the patient. With this prevention, the patient’s chances of dying are reduced, as he or she is less likely to develop severe septic diseases.

From the foregoing, it is observable that the use of telemedicine in the treatment of patients with blood cancer in the Czech Republic is a very appropriate tool for making treatment more effective, both from the patient’s perspective, as well as from the perspective of hospitals, health insurance companies, and the entire health care system, even though the introduction of these modern technologies is associated with a significant initial investment or increased personnel costs for their provision in the initial phase. The return on this investment, as shown by the above analysis, is very short and incomparable with the financial and non-financial benefits for all mentioned stakeholders.
